# Evolution of Indocyanine Green Fluorescence in Breast and Axilla Surgery: An Australasian Experience

**DOI:** 10.3390/life14010135

**Published:** 2024-01-17

**Authors:** Chu Luan Nguyen, Nirmal Dayaratna, Susannah Graham, Farhad Azimi, Cindy Mak, Carlo Pulitano, Sanjay Warrier

**Affiliations:** 1 Department of Breast Surgery, Chris O’Brien Lifehouse, Camperdown, NSW 2050, Australia; susannah@thebreastcarecentre.com.au (S.G.); fred.azimi.54@gmail.com (F.A.); cindy@thebreastcarecentre.com.au (C.M.); sanjay@drsanjaywarrier.com.au (S.W.); 2 Department of Surgery, Royal Prince Alfred Hospital, Camperdown, NSW 2050, Australia; carlo.pulitano@sydney.edu.au; 3 Department of Surgery, The University of Sydney, Camperdown, NSW 2050, Australia; nirmday@gmail.com

**Keywords:** indocyanine green, fluorescence, breast, axilla, mastectomy, reconstruction, sentinel lymph node

## Abstract

The evolution of indocyanine green (ICG) fluorescence in breast and axilla surgery from an Australasian perspective is discussed in this narrative review with a focus on breast cancer and reconstruction surgery. The authors have nearly a decade of experience with ICG in a high-volume institution, which has resulted in publications and ongoing future research evaluating its use for predicting mastectomy skin flap perfusion for reconstruction, lymphatic mapping for sentinel lymph node (SLN) biopsy, and axillary reverse mapping (ARM) for prevention of lymphoedema. In the authors’ experience, routine use of ICG angiography during breast reconstruction postmastectomy was demonstrated to be cost-effective for the reduction of ischemic complications in the Australian setting. A novel tracer combination, ICG–technetium-99m offered a safe and effective substitute to the “gold standard” dual tracer for SLN biopsy, although greater costs were associated with ICG. An ongoing trial will evaluate ARM node identification using ICG fluorescence during axillary lymph node dissection and potential predictive factors of ARM node involvement. These data add to the growing literature on ICG and allow future research to build on this to improve understanding of the potential benefits of fluorescence-guided surgery in breast cancer and reconstruction surgery.

## 1. Introduction

Surgery is mostly guided by the surgeon’s perception of the tissue under optically visible light. Fluorescence-guided surgery is a relatively new field, which could be added to the surgeon’s armamentum, providing additional information about the anatomy, physiology and pathology of the patient intraoperatively [1,2,3,4,5].

There has been a marked increase in the use of fluorescence-guided surgery over the last decade with indocyanine green (ICG) being the most commonly used fluorophore. ICG is a water-soluble dye that binds to albumin, is rapidly distributed in the blood, and is excreted by the liver. ICG can be administered intravenously or interstitially, depending on the required target. ICG fluorescence-guided surgery relies on the ability of ICG to absorb near-infrared (NIR) light at an 800 nm wavelength and subsequently emit fluorescence. The fluorescent image or video is able to be captured by a dedicated camera to provide real-time information for the operator [6].

ICG was first used to measure liver function and cardiac output. Its use then rapidly expanded after the 1970s when improvements in digital imaging occurred to include imaging of vasculature of the brain, retina and heart [1,6,7]. It has since diversified even further to be applicable to other surgical specialties, including upper gastrointestinal and colorectal surgery, as well as surgical oncology [8,9]. Its use in breast cancer and reconstruction surgery is relatively recent, with an increase in its use over the past ten years [10].

ICG fluorescence is a versatile technology within breast cancer and reconstruction surgery. It can be used to evaluate mastectomy skin flap perfusion during breast reconstruction and to map lymphatics to assist with sentinel lymph node (SLN) biopsy for early breast cancer [11,12,13,14,15]. This narrative review examines the evolution of ICG fluorescence in breast and axilla surgery from an Australasian perspective. The research presented adds to the growing literature on ICG and paves the way for future research to build on this to improve understanding of the potential benefits of fluorescence-guided surgery in breast cancer and reconstruction surgery.

## 2. Indocyanine Green in Breast Cancer and Reconstructive Surgery

Breast cancer is the commonest malignancy in females, affecting one in eight during their lifetime and accounting for one-quarter of all cancer cases worldwide [16,17]. Mastectomy with reconstruction, SLN biopsy, and axillary lymph node dissection (ALND) are common and important breast surgeries. They allow for staging and management of breast cancer as well as restoring cosmesis but carry the risk of significant complications. The use of ICG fluorescence during these procedures could potentially reduce complication rates and improve outcomes for breast cancer survivors.

### 2.1. Mastectomy Skin Flap Perfusion Assessment

Breast reconstruction has been demonstrated to significantly improve the quality of life for females who have undergone mastectomy following a cancer diagnosis. It has also become a more common operation, as evidenced by the rising national breast reconstruction rate over the last ten years [18]. This could be due to the improved understanding of breast cancer oncology, more specialized breast surgeons, and increased advocacy by breast cancer organizations recognizing the advantages of breast reconstruction in patients with cancer. The success of the procedure, however, is limited by the major complication of mastectomy skin flap necrosis (MSFN), which has a reported prevalence of up to 24% following skin-sparing and nipple-sparing breast reconstruction [19,20]. Mastectomy skin flap necrosis can potentially be a devastating complication with attendant risks of infection, implant loss, reoperation, delayed adjuvant systemic therapy, poor cosmesis and the associated financial costs [21]. Skin perfusion is, therefore, a critical principle to the success of breast reconstruction surgery. Clinical judgment has long been the most widely used technique for assessing skin flap perfusion intraoperatively, but it is subjective and relies on surgeon experience [20].

ICG angiography has been recently used to evaluate the skin flap intraoperatively in breast reconstruction surgery in an effort to minimize the subjectivity of perfusion analysis using clinical judgment [22,23]. Real-time fluorescence imaging utilizing a NIR camera after intravenous administration of ICG allows for an angiogram to be produced and viewed intraoperatively. ICG has a short plasma half-life (three to four minutes) and an excellent safety profile with a very low incidence of adverse events (1 out of 42,000 patients), which allows for multiple uses during the same operation safely [10,24,25]. The angiograms provide a dynamic map of dermal circulation for tissue perfusion analysis by the operator, as well as the ability to quantitatively measure perfusion using the integrated software. This potentially makes this technique less subjective compared to traditional clinical evaluation on its own [26].

Tissue dissection during mastectomy is inherently accompanied by devascularization. Accurate evaluation of tissue viability is critical as early identification of poorly perfused tissue could help guide intraoperative decision-making, such as the need for flap revision or a delayed procedure to mitigate the risk of postoperative ischemic complications [27]. Although studies have shown that ICG angiography may reliably predict postoperative skin flap necrosis, there are currently no randomized controlled trials (RCTs) published to validate this technique [23]. Furthermore, there is no consensus yet on the optimal ICG angiography protocol and which parameters of the integrated software to use to determine tissue at risk of necrosis, which has limited its widespread use. Evaluating the effectiveness and standardizing the methodology of ICG angiography could provide surgeons with a more reliable tool to predict necrosis intraoperatively.

#### 2.1.1. Utility of Indocyanine Green Angiography in Delaying Breast Reconstruction Postmastectomy

Adequate skin perfusion following skin-sparing mastectomy (SSM) and nipple-sparing mastectomy (NSM) is key to avoiding ischemic complications [23,28,29]. Previous studies have demonstrated that ICG angiography can provide useful information to help intraoperative decision-making involving the excision of ischemic areas of mastectomy skin flaps. The diagnostic accuracy of ICG angiography for predicting necrosis has also been investigated, but only in small cohorts [30,31,32,33,34,35].

The authors have evaluated a more conservative approach in which cases identified with poorly perfused mastectomy skin flaps had their implant-based breast reconstruction delayed to a separate admission. The authors have assessed the utility of ICG angiography in the decision to delay reconstruction and its diagnostic accuracy over six years of its routine use in a large cohort [13,36]. Patients with adequate ICG perfusion analysis received immediate reconstruction with an implant or tissue expander. In those with inadequate ICG perfusion, the mastectomy pocket was left empty, and the skin closed while awaiting a delayed reconstruction. Identification of poorly perfused tissue using ICG angiography was shown in this study to help guide the intraoperative decision to delay reconstruction of 61 of 320 total reconstructions (19%), which did not result in any cases of necrosis in the delayed reconstruction cohort. The mean intraoperative ICG angiography perfusion values immediately following mastectomy of these delayed reconstructions were significantly reduced compared to the values recorded at their delayed reconstruction procedures. Patients awaiting a delayed reconstruction did not receive any additional treatment to try to improve tissue perfusion. Delaying the reconstruction by a median of 7.3 days yielded improved perfusion values, which were adequate to mitigate the risk of skin flap necrosis. Routine use of ICG angiography over six years was found to be a useful adjunct to clinical assessment in flap perfusion evaluation.

#### 2.1.2. Indocyanine Green Angiogram Patterns of Ischemia and Reperfusion in Nipple-Sparing Mastectomy Reconstruction

Nipple-sparing mastectomy combines the SSM technique with preservation of the nipple–areolar complex (NAC) to improve cosmesis. The operation is associated with acceptable oncological outcomes with low local recurrence rates of 3–6% at five years, which are comparable to rates of up to 4.9% with traditional mastectomy [37,38]. Nipple-sparing mastectomy, however, carries a higher risk of MSFN as well as nipple necrosis. This is related to the attempted preservation of a skin envelope with a greater surface area that requires adequate perfusion. Known risk factors that can compromise skin flap perfusion include smoking, diabetes, obesity, large breasts and radiotherapy [39,40]. Knowledge of such patient-related factors assists with patient selection and better-informed decision-making. Patterns of ischemia of mastectomy flaps, afforded by intraoperative ICG angiography, could potentially assist with skin flap perfusion assessment and improve decision-making during surgery to reduce the risk of postoperative necrosis.

**Figure 1:**
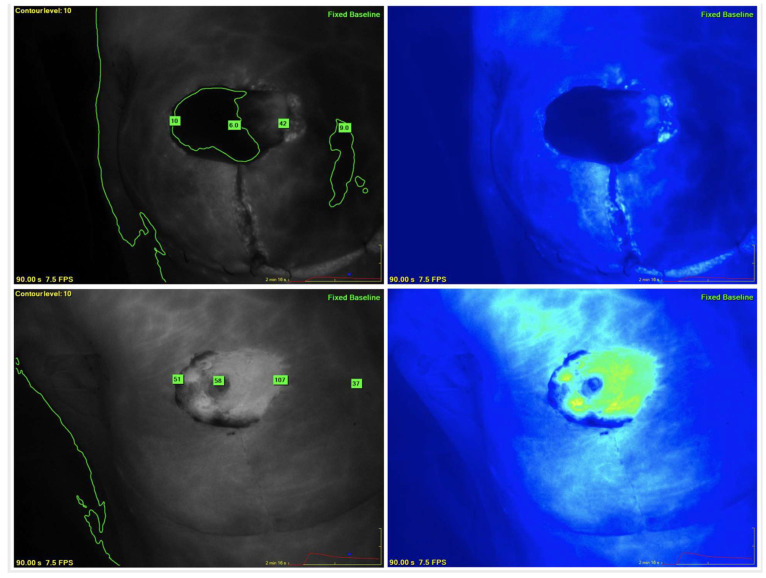
Indocyanine green (ICG) angiograms with fluorescence patterns of ischemia showing nipple-areolar-complex (NAC) ischemia with reperfusion one week later. (**Top left**): grey-scale ICG angiogram immediately following nipple-sparing mastectomy with contour level set at 10 units and absolute perfusion units applied at the NAC and medial edge of skin flap; (**Top right**): colorized ICG angiogram following NSM; (**Bottom left and right**): angiograms of the same breast shot just prior to delayed breast reconstruction.

Nipple-sparing mastectomy reconstruction can be completed as single or multistage procedures. Immediate breast reconstruction following mastectomy has been demonstrated to be associated with higher postoperative necrosis rates compared with multistage procedures. Immediate reconstructions are understood to be associated with a higher degree of vascular stress and tension on mastectomy skin flaps, which is further compounded by the inability to induce neovascularization to the NAC [40]. The authors have shown that it is possible to identify patients intraoperatively with ischemia of the mastectomy skin flap immediately following NSM based on ICG angiography analysis [12]. The reconstruction surgery for these patients was subsequently delayed after a median of seven days. Most mastectomy skin flaps and NAC had significantly improved perfusion on repeat ICG angiography at the delayed operation. The delayed reconstruction alone, without any other intraoperative intervention, was sufficient to allow reperfusion of the ischemic flaps. The improvement in perfusion was identified on both the fluorescence angiograms and when perfusion units were applied to the skin flaps and NAC using the integrated software (Figure 1) [12].

A reliable classification system of patterns of skin flap and NAC ischemia could allow surgeons to recognize high-risk cases and assist with intraoperative decision-making to reduce the risk of ischemic complications. Further defining types of skin flap ischemia and their severity using quantitative analysis could potentially allow for the development of future algorithms for its management. Ischemic flaps can have different etiologies that manifest different fluorescence patterns. The management of such ischemic flaps could then be tailored by the type of ischemia. ICG angiography could enable more targeted intraoperative decisions, including local debridement or resection of geographic or incisional areas of ischemia prior to reconstruction. Cases identified with diffuse ischemic patterns could be managed with a smaller volume of tissue expander filling or with the use of a smaller implant. Cases with diffuse and severe ischemia could have their entire reconstruction delayed. Another potential advantage of fluorescence patterns could also be to provide real-time feedback to surgeons and assistants regarding their surgical technique, to avoid dissection deep into the dermis, which could thin flaps or cause aggressive retraction on flaps, causing unnecessary trauma. Combining ICG fluorescence images and quantitative analysis with perfusion measurements, as well as clinical judgment, can help with identifying tissue at risk of necrosis.

#### 2.1.3. Trends in Outcomes with Adoption of Indocyanine Green Angiography in Postmastectomy Reconstruction

There is a learning curve when a new surgical technology is introduced, which can be complex and multifactorial, including surgical skills and experience. While evaluating a new technology’s safety and efficacy is important, defining trends in outcomes over time can be useful in determining whether to adopt new technology into clinical practice [41].

Little is known about the learning curve of ICG angiography despite the improved outcomes that have been demonstrated thus far. There could be interuser variation with the use of ICG angiography due to the lack of widespread uptake and consensus on a standardized protocol for the interpretation of its angiograms. There are limited data on whether outcomes improve with increased use of ICG angiography, and the number of cases required to sustain any reduced rate of postoperative ischemic complications [27]. Data on the associated morbidity with adopting this technique could also potentially guide the development of standardized protocols for its use. In an attempt to answer these questions, the authors have evaluated changes in patient outcomes with increasing case volume after ICG angiography adoption in the context of postmastectomy implant-based reconstruction [15].

Outcomes after ICG angiography implementation were identified to have improved over time with increasing case volume at the authors’ institution. Previous studies have reported ICG angiography to be highly sensitive and specific in predicting postoperative MSFN [23,28,30,31,42,43]. The authors used the technology as a supplement with clinical judgment in the assessment of skin flap perfusion for implant-based reconstructions. A marked trend was observed with an increased proportion of delayed reconstructions undertaken over time. The additional perfusion information obtained from ICG angiography contributed towards the decision to delay reconstructions. The aim of this was to mitigate the risk of flap necrosis by reducing the additional insult the flap would have received if an immediate reconstruction was performed. A more cautious approach to immediate reconstruction was utilized over time. This could have influenced the reduction in the rate of ischemic complications, which fell from 27.7% to 2.5% after the implementation of ICG angiography over 320 breast reconstructions, consistent with previous studies [44]. A significant number of cases, however, were needed to reach the plateau of minimal ischemic complications. These data could encourage the development of standardized protocols for the use of ICG angiography to shorten the learning curve for improved patient outcomes.

#### 2.1.4. Cost-Effectiveness of Indocyanine Green Angiography in Postmastectomy Breast Reconstruction

It is also important to evaluate new technologies from a cost-effectiveness perspective, given the significant financial burden of healthcare costs on patients and the healthcare system. This can help to better yield cost savings for the healthcare system. The cost-effectiveness of ICG angiography in breast reconstruction surgery remains in question, although the utility of this technology has been increasing recently. There is a scarcity of cost analysis studies in the current literature, with only a small number of published studies and conflicting data on whether ICG angiography use is cost-effective. These cost analyses have also only been performed in the United States based on their healthcare costs and may not be applicable to other countries [45].

The authors have evaluated the cost-effectiveness of routine ICG angiography use in implant-based breast reconstruction in the Australian context [14]. The findings were that its routine use was a cost-effective intervention for the reduction of ischemic complications. ICG angiography use afforded a gain of 1.77 additional years of perfect health at the cost of AUD 656 more per year, which favored it as a cost-effective intervention. If the technology could be sold at a lower cost in the future, this would make the current break-even number of 318 lower and potentially produce further long-term cost savings. Although ICG angiography was a costly technology to implement, the cost savings from reducing ischemic complications and its sequalae of hospital readmissions, reoperations and implant losses were significantly greater. This information adds to the existing literature on health economics and may assist in the decision to adopt this technology in clinical practice.

### 2.2. Lymphatic Mapping and Sentinel Lymph Node Biopsy for Breast Cancer

**Figure 2:**
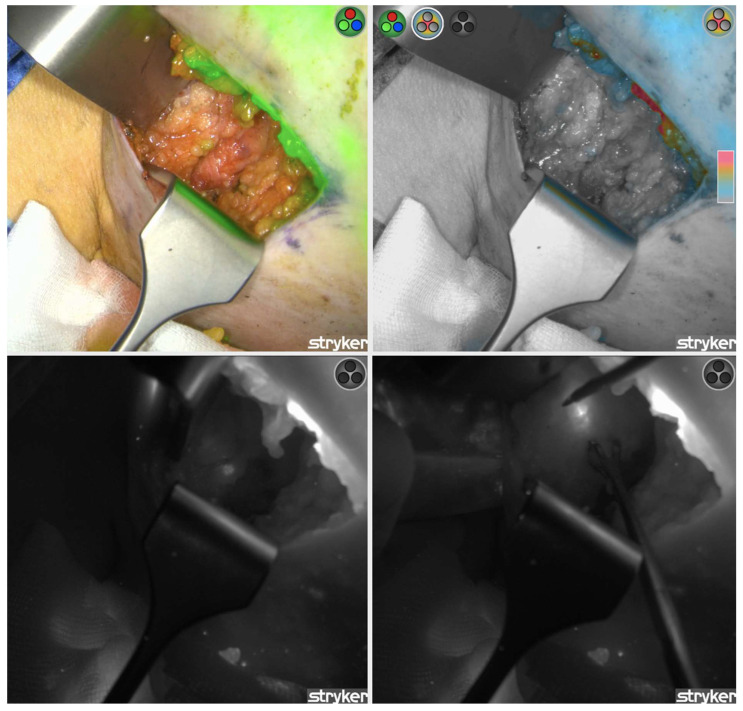
Indocyanine green (ICG) fluorescence-guided sentinel lymph node (SLN) mapping. (**Top left**): Right axillary SLN identification with overlay fluorescence mode; (**Top right**): Color-segmented fluorescence mode; (**Bottom left**): The “green” SLN much more easily identified with fluorescence mode; (**Bottom right**): “Green” SLN dissection with cautery.

Fluorescent visualization of SLNs has been described for melanoma as well as gynecological, gastrointestinal, head and neck, and urological malignancies [2]. Its use for lymphatic mapping and nodal identification in breast cancer is relatively new. ICG fluorescence-guided surgery is similar to ICG angiography in that it uses the same dye and NIR imaging but with some differences in the technique. ICG fluorescence imaging allows the surgeon to visualize subcutaneous lymphatic flow in real time for axillary dissection of SLNs for breast cancer [46,47,48]. The dye binds to plasma albumin and behaves as a fluorescent tracer of lymphatic channels and nodes, which are viewed on a NIR display (Figure 2) [49]. The advantages of this technique are its real-time visualization, visibility through the skin, and excellent safety profile. The limitations, however, include difficulties with the detection of deeper SLNs in larger patients and a higher number of detected nodes compared to traditional tracers, which could predispose to postoperative lymphoedema [50,51].

Gold standard lymphatic mapping for SLNs for biopsy utilizes the combination of blue dye (BD) and technietium-99m (99mTc) but can be complicated by allergic reactions, skin tattooing, radiation and the need for nuclear medicine access [52]. These limitations have led to the development of new techniques, which include ICG fluorescence imaging [50]. ICG fluorescence for SLN biopsy in early breast cancer has been demonstrated in cohort studies to be comparable to a single technique using a radioisotope labeled with 99mTc, and superior to a single technique with BD [50]. There is, however, a lack of RCTs comparing ICG with 99mTc or BD on its own. Evaluating the effectiveness of ICG compared with the gold standard for SLN biopsy could potentially lead to the elimination of the disadvantages associated with traditional techniques. ICG could theoretically be used as a sole tracer agent for SLN biopsy as it combines the key advantages of BD and 99mTc without some of the disadvantages. More data to validate this technique, however, are warranted.

#### 2.2.1. Dual Tracer Indocyanine Green and Radioisotope Compared to Gold Standard Sentinel Lymph Node Biopsy in Breast Cancer

Lymphatic mapping and SLN biopsy with the combination of BD and 99mTc have become a diagnostic standard of care in clinically node-negative early breast cancer within the last two decades [53,54]. It has high SLN detection rates and low false-negative rates of 96.7% and 5.5%, respectively [52,55,56,57,58,59]. A new technique should ideally be as effective as the current standard and at a reasonable cost for it to be justified for clinical practice. The authors have begun to answer this question by comparing the combination of a novel dual tracer, ICG–99mTc, for SLN biopsy in early breast cancer with a control cohort using the traditional combination of BD–99mTc. This was a unique study compared to previous studies, which used fluorescence-guided SLN biopsy in combination with a comparator tracer (99mTc and/or BD) in one cohort [50,51,52,60].

In the authors’ study, BD was replaced with ICG following a decade of BD use. The outcomes of the study demonstrated equivalency between the two dual techniques in terms of the number of SLNs identified, the rate of failed mapping, and the identification of metastatic SLNs. These results are useful as they could aid surgeons who use BD in considering an alternative tracer. The proviso is the higher costs associated with ICG. Abandonment of 99mTc lymphoscintigraphy in the near future is highly unlikely, given how well established it is. Using ICG as an adjunct to 99mTc would be a more reasonable approach to consider, as it can be complementary to 99mTc as a replacement for BD.

#### 2.2.2. Indocyanine Green Compared to Technetium-99m for Sentinel Lymph Node Biopsy in Breast Cancer

The use of 99mTc on its own has a detection rate of 96.5%, and BD on its own has a detection rate of 86.8% [52]. Disadvantages of BD tracer include skin tattooing and hypersensitivity reactions, while those from 99mTc include inconvenience to patients, radiation exposure, and the requirement for nuclear medicine access [61]. The localization technique using ICG fluorescence, however, requires further investigation. So far, in smaller studies, ICG has shown comparable sensitivity to 99mTc, with SLN detection rates ranging from 93.1% to 100% [50,60,62,63,64]. More data from prospective trials evaluating noninferiority as well as cost analysis are required.

A drawback of fluorescence-guided SLN biopsy is that it requires additional costs for the ICG drug, camera, and associated disposable materials. The use of 99mTc also has additional costs from hospital infrastructure to accommodate radioactive materials, specialist staff to perform the lymphoscintigraphy, and patient travel [65]. The authors are currently running a prospective trial to compare the equivalency of ICG directly to 99mTc for axillary SLN lymphatic mapping in early breast cancer in terms of the number of SLNs identified, including metastatic nodes, the rate of failed mapping, ease of use, and associated costs (Registered on ANZCTR: ACTRN12621001033831). So far, 205 patients have been enrolled at the three-quarter mark. There have been no adverse reactions to ICG. Technetium-99m cost an additional AUD 1492.72 per case, but ICG would require over 35 cases before breaking even with initial outlay equipment costs. The trial data so far are promising as they demonstrate that ICG fluorescence is comparable to 99mTc in terms of the number of SLNs identified, including metastatic nodes, and the rate of failed mapping (Table 1). While BD remains the standard of care in Australia, it may not be necessary in the future as more data on ICG become available.

### 2.3. Axillary Reverse Mapping with Indocyanine Green

Breast cancer-related lymphoedema (BCRL) is a common complication after axillary dissection for breast cancer. It negatively affects quality of life due to discomfort and reduced upper limb function. Due to needless sacrifice of lymphatics of the arm during SLN biopsy and ALND procedures, in results in a higher incidence of 14% in ALND compared to 2% in SLNB [66]. The technique of axillary reverse mapping (ARM) can help differentiate lymphatic drainage of the arm from the breast in the axilla. Axillary reverse mapping aims to minimize potential disturbance of arm lymphatics to mitigate the risk of BCRL during axillary dissection [66,67,68]. This involves intradermal injection of BD, 99mTc or ICG to visualize arm lymphatics.

The combination of ALND with ARM has been demonstrated to reduce the rates of BCRL compared to standard procedures on their own [69]. The oncological safety of ARM is a major concern, which has limited its widespread use because arm lymphatic drainage is not completely independent from that of the breast. Crossover “SLN-ARM” nodes are those that share similar lymphatic channels as those for the breast and these have been identified to exist. Preserving these nodes to reduce BCRL, however, could increase the risk of metastases, and so the fine balance between them has yet to be fully elucidated [70,71,72]. A systematic approach of conserving the arm SLNs is currently not recommended due to the high number of metastasis-positive ARM nodes. ARM node preservation could potentially be reserved for select patients who have been estimated to have low axillary involvement preoperatively. Identifying prognostic factors for metastasis to the ARM nodes could allow for algorithms for node preservation during ALND to be developed [73]. Validating this could potentially lead to its widespread use and prevention of BCRL while being oncologically safe.

Axillary reverse mapping has been studied for a decade since its introduction by Thompson and Nos et al. [74,75]. Significant issues, particularly with the low identification rate, the effectiveness of reducing arm lymphoedema, and oncological safety, remain unresolved. The authors are currently running a prospective trial that aims to evaluate ARM node identification using ICG fluorescence during ALND and predictive factors of ARM node involvement. Enrolled patients have breast cancer with an indication for ALND. ICG fluorescence is utilized during standard ALND to distinguish ARM nodes and their corresponding lymphatic ducts. Excised ARM lymph nodes are isolated from the other axillary lymph nodes and sent to histopathology separately for analysis (Trial registered on ANZCTR: ACTRN12621000817842).

Lymphoedema rates have the potential to be reduced if the current issues with ARM can be solved. There is potential for even more benefits if the ARM technique can be successfully combined with lymphovenous bypass techniques. Lymphovenous anastomosis aims to overcome localized obstructions in the lymphatics associated with ALND by diverting lymph into the venous system from distended lymphatics proximal to obstructed areas [3,71]. This is an area that the authors are interested in, and there are plans to investigate this in the near future.

## 3. Conclusions and Future Perspectives

ICG fluorescence-guided surgery is an innovative technique that is applicable to numerous surgical specialities. The potential indications for its use in breast cancer and reconstruction surgery are diverse. The evolution of indocyanine green (ICG) fluorescence in breast and axilla surgery from an Australasian perspective was discussed in this narrative review. The authors have had nearly a decade of experience with ICG in a high-volume institution, which has resulted in publications and ongoing future research evaluating its use for predicting skin flap necrosis for reconstruction, lymphatic mapping for SLN biopsy, and ARM for prevention of lymphoedema. The challenge for the future will be trials to further validate this technology to justify its incorporation into routine clinical practice. Given the present experience and future opportunities, ICG fluorescence is a promising technology.

## Figures and Tables

**Table 1:** Preliminary results of indocyanine green compared with technetium-99m for sentinel lymph node biopsy in early breast cancer, N = 205 patients, 450 sentinel lymph nodes.

	ICG	99mTc	*p*
Mean number of SLNs (SD)	2.1 (2.14)	2.2 (2.17)	0.85
Failed mapping, N (%)	15 (3.3)	10 (2.2)	0.36
Metastatic SLNs, N (%)	57 (96.6)	58 (98.3)	0.32
Ease of detection *, mean (SD)	1.6 (0.93)	1.5 (0.64)	0.1

ICG, indocyanine green; 99mTc, technetium-99m; SD, standard deviation; SLN, sentinel lymph node; * Likert scale, 1 = very easy, 2 = easy, 3 = equivocal, 4 = hard, 5 = very hard.

## Data Availability

Data are available on request to the corresponding author.

## References

[1] Dai Z.Y., Shen C., Mi X.Q., Pu Q. (2023). The primary application of indocyanine green fluorescence imaging in surgical oncology. Front. Surg..

[2] Sutton P.A., van Dam M.A., Cahill R.A., Mieog S., Polom K., Vahrmeijer A.L., van der Vorst J. (2023). Fluorescence-guided surgery: Comprehensive review. BJS Open.

[3] Vidya R., Leff D.R., Green M., McIntosh S.A., St John E., Kirwan C.C., Romics L., Cutress R.I., Potter S., Carmichael A. (2021). Innovations for the future of breast surgery. Br. J. Surg..

[4] Xia S., Zhang Y., Fang M., Mikesell L., Steenwinkel T.E., Wan S., Phillips T., Luck R.L., Werner T., Liu H. (2019). A FRET-Based Near-Infrared Fluorescent Probe for Ratiometric Detection of Cysteine in Mitochondria. ChemBioChem.

[5] Zhang Y., Sun L., Yan Q., Qiu X., Cheng Y., Wang B., Tan X., Fang M., Luck R.L., Liu H. (2022). Near-infrared fluorescent probe based on cyanine scaffold for sensitive detection of uranyl ions in living cells and water samples. Microchem. J..

[6] Reinhart M.B., Huntington C.R., Blair L.J., Heniford B.T., Augenstein V.A. (2016). Indocyanine green: Historical context, current applications, and future considerations. Surg. Innov..

[7] Yannuzzi L.A. (2011). Indocyanine green angiography: A perspective on use in the clinical setting. Am. J. Ophthalmol..

[8] Kawakita N., Takizawa H., Sawada T., Matsumoto D., Tsuboi M., Toba H., Yoshida M., Kawakami Y., Kondo K., Tangoku A. (2019). Indocyanine green fluorescence imaging for resection of pulmonary metastasis of hepatocellular carcinoma. J. Thorac. Dis..

[9] Pameijer C.R., Leung A., Neves R.I., Zhu J. (2018). Indocyanine green and fluorescence lymphangiography for sentinel node identification in patients with melanoma. Am. J. Surg..

[10] Griffiths M., Chae M.P., Rozen W.M. (2016). Indocyanine green-based fluorescent angiography in breast reconstruction. Gland Surg..

[11] Nguyen C.L., Zhou M., Easwaralingam N., Seah J.L., Azimi F., Mak C., Pulitano C., Warrier S. (2023). Novel Dual Tracer Indocyanine Green and Radioisotope versus Gold Standard Sentinel Lymph Node Biopsy in Breast Cancer: The GREENORBLUE Trial. Ann. Surg. Oncol..

[12] Nguyen C.L., Tam S.K.M., Easwaralingam N., Seah J.L., Comerford A.P., Yu A.C.X., Mak C., Pulitano C., Warrier S.K. (2022). Patterns of ischaemia and reperfusion in nipple-sparing mastectomy reconstruction with indocyanine green angiography. J. Plast. Reconstr. Aesthetic Surg..

[13] Nguyen C.L., Liu A., Lata T., Easwaralingam N., Seah J.L., Chan C., Cao F., Azimi F., Mak C., Pulitano C. (2022). Utility of indocyanine green angiography in delaying breast reconstruction postmastectomy. Eur. J. Plast. Surg..

[14] Nguyen C.L., Dayaratna N., Comerford A.P., Tam S.K.M., Paredes S.R., Easwaralingam N., Seah J.L., Azimi F., Mak C., Pulitano C. (2022). Cost-effectiveness of indocyanine green angiography in postmastectomy breast reconstruction. J. Plast. Reconstr. Aesthetic Surg..

[15] Nguyen C.L., Comerford A.P., Dayaratna N., Lata T., Paredes S.R., Easwaralingam N., Seah J.L., Azimi F., Mak C., Pulitano C. (2022). Trends in outcomes with adoption of indocyanine green angiography in postmastectomy reconstruction. ANZ J. Surg..

[16] Bray F., Ferlay J., Soerjomataram I., Siegel R.L., Torre L.A., Jemal A. (2012). Global cancer statistics 2018: GLOBOCAN estimates of incidence and mortality worldwide for 36 cancers in 185 countries. CA Cancer J. Clin..

[17] Ferlay J., Soerjomataram I., Dikshit R., Eser S., Mathers C., Rebelo M., Parkin D.M., Forman D., Bray F. (2015). Cancer incidence and mortality worldwide: Sources, methods and major patterns in GLOBOCAN 2012. Int. J. Cancer.

[18] Dayaratna N., Nguyen C.L., Spillane A., Mak C., Warrier S.K., Dusseldorp J.R. (2023). Trends and variations in post-mastectomy breast reconstruction rates in Australia over 10 years. ANZ J. Surg..

[19] Woerdeman L.A.E., Hage J.J., Smeulders M.J.C., Rutgers E.J.T., van der Horst C.M.A.M. (2006). Skin-Sparing Mastectomy and Immediate Breast Reconstruction by Use of Implants: An Assessment of Risk Factors for Complications and Cancer Control in 120 Patients. Plast. Reconstr. Surg..

[20] Kobraei E.M., Nimtz J., Wong L., Buseman J., Kemper P., Wright H., Rinker B.D. (2012). Risk factors for adverse outcome following skin-sparing mastectomy and immediate prosthetic reconstruction. Plast. Reconstr. Surg..

[21] Jeon F.H.K., Varghese J., Griffin M., Butler P.E., Ghosh D., Mosahebi A. (2018). Systematic review of methodologies used to assess mastectomy flap viability. BJS Open.

[22] Singer R., Lewis C.M., Franklin J.D., Lynch J.B. (1978). Fluorescein test for prediction of flap viability during breast reconstructions. Plast. Reconstr. Surg..

[23] Pruimboom T., Schols R.M., Van Kuijk S.M., Van der Hulst R.R., Qiu S.S. (2020). Indocyanine green angiography for preventing postoperative mastectomy skin flap necrosis in immediate breast reconstruction. Cochrane Database Syst. Rev..

[24] Benya R., Quintana J., Brundage B. (1989). Adverse reactions to indocyanine green: A case report and a review of the literature. Catheter. Cardiovasc. Diagn..

[25] Hope-Ross M., Yannuzzi L.A., Gragoudas E.S., Guyer D.R., Slakter J.S., Sorenson J.A., Krupsky S., Orlock D.A., Puliafito C.A. (1994). Adverse Reactions due to Indocyanine Green. Ophthalmology.

[26] Muntean M.V., Ardelean F., Strilciuc S., Pestean C., Georgescu A.V., Muntean V. (2019). Flap warming improves intraoperative indocyanine green angiography (ICGA) assessment of perfusion. An experimental study. J. Plast. Reconstr. Aesthetic Surg..

[27] Diep G.K., Marmor S., Kizy S., Huang J.L., Jensen E.H., Portschy P., Cunningham B., Choudry U., Tuttle T.M., Hui J.Y.C. (2019). The use of indocyanine green angiography in postmastectomy reconstruction: Do outcomes improve over time?. J. Plast. Reconstr. Aesthetic Surg..

[28] Komorowska-Timek E., Gurtner G.C. (2010). Intraoperative perfusion mapping with llaser-assisted indocyanine green imaging can predict and prevent complications in immediate breast reconstruction. Plast. Reconstr. Surg..

[29] Driessen C., Arnardottir T.H., Lorenzo A.R., Mani M.R. (2020). How should indocyanine green dye angiography be assessed to best predict mastectomy skin flap necrosis? A systematic review. J. Plast. Reconstr. Aesthetic Surg..

[30] Phillips B.T., Lanier S.T., Conkling N., Wang E.D., Dagum A.B., Ganz J.C., Khan S.U., Bui D.T. (2012). Intraoperative perfusion techniques can accurately predict mastectomy skin flap necrosis in breast reconstruction: Results of a prospective trial. Plast. Reconstr. Surg..

[31] Munabi N.C., Olorunnipa O.B., Goltsman D., Rohde C.H., Ascherman J.A. (2014). The ability of intra-operative perfusion mapping with laser-assisted indocyanine green angiography to predict mastectomy flap necrosis in breast reconstruction: A prospective trial. J. Plast. Reconstr. Aesthetic Surg..

[32] Johnson A.C., Colakoglu S., Chong T.W., Mathes D.W. (2020). Indocyanine Green Angiography in Breast Reconstruction: Utility, Limitations, and Search for Standardization. Plast. Reconstr. Surg. Glob. Open.

[33] Wang C.Y., Wang C.H., Tzeng Y.S., Lin C.T., Chou C.Y., Chiang I.H., Wu C.J., Chen S.G. (2018). Intraoperative Assessment of the Relationship between Nipple Circulation and Incision Site in Nipple-Sparing Mastectomy with Implant Breast Reconstruction Using the SPY Imaging System. Ann. Plast. Surg..

[34] Phillips B.T., Fourman M.S., Rivara A. (2014). Comparing quantitative values of two generations of laser-assisted indocyanine green dye angiography systems: Can we predict necrosis?. Eplasty.

[35] Newman M.I., Jack M.C., Samson M.C. (2013). SPY-Q analysis toolkit values potentially predict mastectomy flap necrosis. Ann. Plast. Surg..

[36] Warrier S., Nguyen C.L., Easwaralingam N. (2021). Acellular dermal matrices in breast reconstruction: A narrative review and institutional perspective. Ann. Breast Surg..

[37] Karian L.S., Therattil P.J., Wey P.D., Nini K.T. (2017). Delay techniques for nipple-sparing mastectomy: A systematic review. J. Plast. Reconstr. Aesthetic Surg..

[38] De La Cruz L., Moody A.M., Tappy E.E., Blankenship S.A., Hecht E.M. (2015). Overall Survival, Disease-Free Survival, Local Recurrence, and Nipple-Areolar Recurrence in the Setting of Nipple-Sparing Mastectomy: A Meta-Analysis and Systematic Review. Ann. Surg. Oncol..

[39] Mastroianni M., Lin A.M., Smith B.L., Austen W.G., Colwell A.S. (2016). Nipple Loss following Nipple-Sparing Mastectomy. Plast. Reconstr. Surg..

[40] Tondu T., Hubens G., Tjalma W.A., Thiessen F.E., Vrints I., Van Thielen J., Verhoeven V. (2020). Breast reconstruction after nipple-sparing mastectomy in the large and/or ptotic breast: A systematic review of indications, techniques, and outcomes. J. Plast. Reconstr. Aesthetic Surg..

[41] Hopper A.N., Jamison M.H., Lewis W.G. (2007). Learning curves in surgical practice. Postgrad. Med. J..

[42] Moyer H.R., Losken A. (2012). Predicting mastectomy skin flap necrosis with indocyanine green angiography: The gray area defined. Plast. Reconstr. Surg..

[43] Mattison G.L., Lewis P.G., Gupta S.C., Kim H.Y. (2016). SPY Imaging Use in Postmastectomy Breast Reconstruction Patients: Preventative or Overly Conservative?. Plast. Reconstr. Surg..

[44] Duggal C.S., Madni T., Losken A. (2014). An outcome analysis of intraoperative angiography for postmastectomy breast reconstruction. Aesthetic Surg. J..

[45] Nguyen C.L., Barry N., Lindsay A.P., Seah J.L., Easwaralingam N., Pulitano C., Kumar S.K. (2021). Indocyanine green angiography in breast reconstruction surgery: A systematic review of cost-analysis studies. J. Plast. Reconstr. Aesthetic Surg..

[46] Kusano M., Tajima Y., Yamazaki K., Kato M., Watanabe M., Miwa M. (2008). Sentinel node mapping guided by indocyanine green fluorescence imaging: A new method for sentinel node navigation surgery in gastrointestinal cancer. Dig. Surg..

[47] Korn J.M., Tellez-Diaz A., Bartz-Kurycki M., Gastman B. (2014). Indocyanine green SPY elite-assisted sentinel lymph node biopsy in cutaneous melanoma. Plast. Reconstr. Surg..

[48] Sugie T., Kinoshita T., Masuda N., Sawada T., Yamauchi A., Kuroi K., Taguchi T., Bando H., Yamashiro H., Lee T. (2016). Evaluation of the Clinical Utility of the ICG Fluorescence Method Compared with the Radioisotope Method for Sentinel Lymph Node Biopsy in Breast Cancer. Ann. Surg. Oncol..

[49] Benson R.C., Kues H.A. (1978). Fluorescence properties of indocyanine green as related to angiography. Phys. Biol..

[50] Kedrzycki M.S., Leiloglou M., Ashrafian H., Jiwa N., Thiruchelvam P.T.R., Elson D.S., Leff D.R. (2020). Meta-analysis Comparing Fluorescence Imaging with Radioisotope and Blue Dye-Guided Sentinel Node Identification for Breast Cancer Surgery. Ann. Surg. Oncol..

[51] Mok C.W., Tan S.M., Zheng Q., Shi L. (2019). Network meta-analysis of novel and conventional sentinel lymph node biopsy techniques in breast cancer. BJS Open.

[52] Cykowska A., Marano L., D’Ignazio A., Marrelli D., Swierblewski M., Jaskiewicz J., Roviello F., Polom K. (2020). New technologies in breast cancer sentinel lymph node biopsy; from the current gold standard to artificial intelligence. Surg. Oncol..

[53] Donker M., van Tienhoven G., Straver M.E., Meijnen P., van de Velde C.J.H., Mansel R.E., Cataliotti L., Westenberg A.H., Klinkenbijl J.H.G., Orzalesi L. (2014). Radiotherapy or surgery of the axilla after a positive sentinel node in breast cancer (EORTC 10981-22023 AMAROS): A randomised, multicentre, open-label, phase 3 non-inferiority trial. Lancet Oncol..

[54] Mansel R.E., Fallowfield L., Kissin M., Goyal A., Newcombe R.G., Dixon J.M., Yiangou C., Horgan K., Bundred N., Monypenny I. (2006). Randomized multicenter trial of sentinel node biopsy versus standard axillary treatment in operable breast cancer: The ALMANAC Trial. J. Natl. Cancer Inst..

[55] Giuliano A.E., Kirgan D.M., Guenther J.M., Morton D.L. (1994). Lymphatic mapping and sentinel lymphadenectomy for breast cancer. Ann. Surg..

[56] Krag D.N., Weaver D.L., Alex J.C., Fairbank J.T. (1993). Surgical resection and radiolocalization of the sentinel lymph node in breast cancer using a gamma probe. Surg. Oncol..

[57] Lyman G.H., Guiliano A.E., Somerfield M.R. (2005). The American Society of Clinical Oncology Guideline recommendations for sentinel lymph node biopsy in early stage breast cancer. J. Clin. Oncol..

[58] Krag D.N., Anderson S.J., Julian T.B., Brown A.M., Harlow S.P., Costantino J.P., Ashikaga T., Weaver D.L., Mamounas E.P., Jalovec L.M. (2010). Sentinel-lymph-node resection compared with conventional axillary-lymph-node dissection in clinically node-negative patients with breast cancer: Overall survival findings from the NSABP B-32 randomised phase 3 trial. Lancet Oncol..

[59] Efron P., Knudsen E., Hirshorn S., Copeland E.M. (2002). Anaphylactic reaction to isosulfan blue used for sentinel node biopsy: Case report and literature review. Breast J..

[60] Sugie T., Ikeda T., Kawaguchi A., Shimizu A., Toi M. (2017). Sentinel lymph node biopsy using indocyanine green fluorescence in early-stage breast cancer: A meta-analysis. Int. J. Clin. Oncol..

[61] Montgomery L.L., Thorne A.C., Van Zee K.J., Fey J., Heerdt A.S., Gemignani M. (2002). Isosulfan blue dye reactions during sentinel lymph node mapping for breast cancer. Anesth. Analg..

[62] Papathemelis T., Jablonski E., Scharl A., Hauzenberger T., Gerken M., Klinkhammer-Schalke M., Hipp M., Scharl S. (2018). Sentinel Lymph Node Biopsy in Breast Cancer Patients by Means of Indocyanine Green Using the Karl Storz VITOM(R) Fluorescence Camera. BioMed Res. Int..

[63] Shen S., Xu Q., Zhou Y. (2018). Comparison of sentinel lymph node biopsy guided by blue dye with or without indocyanine green in early breast cancer. J. Surg. Oncol..

[64] Bargon C.A., Huibers A., Young-Afat D.A., Jansen B.A.M., Borel-Rinkes I.H.M., Lavalaye J., van Slooten H.J., Verkooijen H.M., van Swol C.F.P., Doeksen A. (2022). Sentinel Lymph Node Mapping in Breast Cancer Patients through Fluorescent Imaging Using Indocyanine Green: The INFLUENCE Trial. Ann. Surg..

[65] Cattin F., Fogacci T., Frisoni G., Fabiocchi L., Dellachiesa L., Semprini G., Samorani D. (2017). ICG Versus 99tc in Breast Surgery-How to Match Quality Health Care and Costs Reduction: A Cost Effectiveness Study. J. Cancer Sci. Ther..

[66] Wijaya W.A., Peng J., He Y., Chen J., Cen Y. (2020). Clinical application of axillary reverse mapping in patients with breast cancer: A systematic review and meta-analysis. Breast.

[67] McEvoy M.P., Ravetch E., Patel G., Fox J., Feldman S. (2021). Prevention of Breast Cancer-Related Lymphedema. Clin. Breast Cancer.

[68] Abbaci M., Conversano A., De Leeuw F., Laplace-Builhe C., Mazouni C. (2019). Near-infrared fluorescence imaging for the prevention and management of breast cancer-related lymphedema: A systematic review. Eur. J. Surg. Oncol..

[69] Schunemann E., Doria M.T., Silvestre J.B., Gasperin P., Cavalcanti T.C., Budel V.M. (2014). Prospective study evaluating oncological safety of axillary reverse mapping. Ann. Surg. Oncol..

[70] Noguchi M., Noguchi M., Nakano Y., Ohno Y., Kosaka T. (2012). Axillary reverse mapping using a fluorescence imaging system in breast cancer. J. Surg. Oncol..

[71] Yuan Q., Wu G., Xiao S.Y., Hou J., Ren Y., Wang H., Wang K., Zhang D. (2019). Identification and Preservation of Arm Lymphatic System in Axillary Dissection for Breast Cancer to Reduce Arm Lymphedema Events: A Randomized Clinical Trial. Ann. Surg. Oncol..

[72] Shao X., Sun B., Shen Y. (2019). Axillary reverse mapping (ARM): Where to go. Breast Cancer.

[73] Conversano A., Abbaci M., Karimi M., Mathieu M.C., de Leeuw F., Michiels S., Laplace-Builhe C., Mazouni C. (2022). Axillary reverse mapping using near-infrared fluorescence imaging in invasive breast cancer (ARMONIC study). Eur. J. Surg. Oncol..

[74] Nos C., Lesieur B., Clough K.B., Lecuru F. (2007). Blue dye injection in the arm in order to conserve the lymphatic drainage of the arm in breast cancer patients requiring an axillary dissection. Ann. Surg. Oncol..

[75] Thompson M., Korourian S., Henry-Tillman R., Adkins L., Mumford S., Westbrook K.C., Klimberg V.S. (2007). Axillary reverse mapping (ARM): A new concept to identify and enhance lymphatic preservation. Ann. Surg. Oncol..

